# Non-collagen genes role in digenic Alport syndrome

**DOI:** 10.1186/s12882-019-1258-5

**Published:** 2019-02-26

**Authors:** S. Daga, C. Fallerini, S. Furini, C. Pecoraro, F. Scolari, F. Ariani, M. Bruttini, M. A. Mencarelli, F. Mari, A. Renieri, A. M. Pinto

**Affiliations:** 1 Medical Genetics Unit, University of Siena, Policlinico Le Scotte, Viale Bracci, 2, 53100 Siena, Italy; 2 Genetica Medica, Azienda Ospedaliera Universitaria Senese, Siena, Italy; 3 Department of Medical Biotechnologies, University of Siena, Siena, Italy; 4 Pediatric Nephrology Unit, Santobono-Pausilipon Hospital, Naples, Italy; 5 Department of Nephrology, University of Brescia, Brescia, Italy

**Keywords:** Alport syndrome, *LAMA5*, *NPHS2*, Digenic inheritance, WES

## Abstract

**Background:**

Alport syndrome is a clinically heterogeneous nephropathy characterized by severe symptomatology at kidney level due to ultrastructural lesions of the glomerular basement membrane (GBM) as consequence of mutations in COL4 genes. The disease has been linked to *COL4A3*/*COL4A4*/*COL4A5* mutations, which impair GBM functionality and can be inherited in a dominant, recessive or X-linked transmission. Although a targeted Next Generation Sequencing approach has allowed identifying families with pathogenic mutations in more than one COL4 α3-α4-α5 heterotrimer encoding genes, leading to conclude for a digenic pattern of inheritance, the role of non-collagen genes in digenic Alport syndrome has not yet been established.

**Methods:**

We employed a whole-exome sequencing approach on three families in whom a digenic pattern of transmission could be suspected because of a likely biparental contribution or an unexplained phenotype in the proband.

**Results:**

We identified in the three probands hypomorphic *LAMA5* mutations co-inherited with pathogenic *COL4* α4-α5 chains mutations. Segregation analysis revealed that the combination of *LAMA5*/*COL4* variants co-segregate with a fully penetrant phenotype in line with a digenic inheritance.

In one of the three probands an hypomorphic variant in *NPHS2* was also found, suggesting that role of other kidney disease related-genes as modifiers.

**Conclusion:**

These findings validate the impact of *LAMA5* mutations in digenic ATS and highlight the role of extracellular matrix’s genes, basement membrane, slit diaphragm and podocyte cytoskeleton in ATS. This underline the need for a more extensive panel approach in the presence of a digenic ATS, in order to better define clinical severity and recurrence risk for family members.

**Electronic supplementary material:**

The online version of this article (10.1186/s12882-019-1258-5) contains supplementary material, which is available to authorized users.

## Background

Alport syndrome (ATS) initially described as a likely autosomal dominant condition, with males being more severely affected than females, [1–5] has been redefined as an X-linked semi-dominant condition [6, 7] after the discovery in 1990 of the causative gene *COL4A5* on the X chromosome (Xq22.3). With time it has become evident that ATS is a genetically heterogeneous disorder for which all three main models of Mendelian inheritance, namely X-linked (XL), autosomal recessive (AR) and autosomal dominant (AD), are applicable [8]. ATS is characterized by clinical heterogeneity and extreme intrafamilial phenotypic variability including the degree of proteinuria, the onset of Chronic Renal Failure (CRF), the progression-rate to End Stage Renal Disease (ESRD), as well as the ocular and hearing involvement. While X-linked semidominant ATS is a well-established entity associated with mutations in *COL4A5*, AR inheritance pattern is associated with two mutations, in either *COL4A3* or *COL4A4* [7, 9] located on chromosome 2(2q35-q37) [8, 10].

The existence of an AD form of ATS (ADAS) distinct from the thin basement membrane nephropathy (TBMN) is sometime debated; however, already in 2000, van der Loop and colleagues provided convincing evidence in favor of the existence of ADAS, identifying a heterozygous mutation in the *COL4A3* gene in a large ATS family from Northern Ireland [11]. Shortly afterwards, some ADAS pedigrees have also been reported by our group [9] and we have then provided clear evidence of the existence of an AD form accounting for about a 30% of cases [12]. Recently, we have reported a digenic pattern of inheritance for ATS demonstrating that the severity of renal involvement correlates with the number of the collagen type IV mutated molecules [13, 14]. However, the digenic model based on the combination of collagen IV mutations partially explains the phenotypic variability. A recent report underlined the contribution of *LAMA5* gene mutations co-segregating with *COL4A5* mutations in patients with a phenotypic spectrum including hematuria, proteinuria, focal segmental glomerulosclerosis, loss of kidney function and renal cortical cysts [15].

Here, we selected patients with a likely digenic pattern of inheritance who presented a phenotype more severe than what expected for a dominant ATS or for whom family history was suggestive of a biparental inheritance and, using a whole-exome sequencing approach, we investigated whether variations in other genes of the extracellular matrix (basement membrane and slit diaphragm) or podocyte cytoskeleton are implicated in the intrafamilial phenotypic variability. We found that hypomorphic heterozygous variants in *LAMA5* encoding for the alpha 5 subunit of laminin 511 segregate with the severity of renal involvement sometimes along with hypomorphic heterozygous variants in other kidney disease-related genes and we concluded for more complex mechanisms of inheritance in which non-collagen genes may play a pivotal role in disease pathogenesis.

## Methods

### Patients enrollment

Genetic counselling was performed at the Medical Genetics Unit in Siena (Azienda Ospedaliera Universitaria Senese, AOUS) where patients with clinical criteria suggestive of ATS, namely a positive family history and/or a indicative kidney biopsy, high tone sensorineural hearing loss, lenticonus and macular flecks were selected for mutation screening in the *COL4A3*, *COL4A4* and *COL4A5* genes. Families underwent genetic counseling and blood samples from the probands and from their family members were collected in EDTA-containing tubes. Patients and healthy family members provided and signed a written informed consent at for the use of DNA samples for diagnostic purposes.

### Whole exome sequencing analysis

Paired-end exome sequencing on probands’ DNA samples was performed by Ion Proton technology (Life Technology) and the mutational analysis was carried on using IonTorrent Suite software (v.5.8.0), while post-run analysis was conducted using Torrent Variant Caller plug-in (v5.8.0.19). External datasets, such as 1000 genomes and ExAC, were used to define novel variants, not previously identified. Variants were filtered for QD (Quality by Depth) using a cutoff of 20 reads to minimize false positive detection. All variants were annotated using 6 different annotation tools, namely SIFT, Polyphen, Mutation Taster, Mutation Assessor, FATHMM/GERP^++^ combined algorithm and LRT, to determine if the substitution was predicted to be deleterious to protein function. For *COL4A3*, *COL4A4* and *COL4A5* genes, according to published guidelines [16] pathogenicity was ascertained if the following criteria were met: non-polymorphic missense mutations or in-frame deletions involving key amino acids, such as glycine in the collagen Gly-X-Y triple helical domain, splice-site mutations and truncating mutations. Pathogenicity of non-synonymous variations other than Gly substitutions was predicted using Alamut software V.2.11 (Interactive Biosoftware, Rouen, France). To determine whether the identified sequence variants were novel or had been previously reported, we searched in the dbSNP database (http://www.ncbi.nlm.nih.gov/projects/SNP/), the Human Gene Mutation Database (http://www.hgmd.cf.ac.uk/ac/index.php), the Leiden Open Variation Database V.3.0 Build 35 (http://www.lovd.nl/3.0/home) and the ALPORT (*COL4A5*) database (http://www.arup.utah.edu/database/ALPORT/ALPORT_display.php?sort = 2#alport; last update: December 2017). Three pathogenic variants were described according to the *COL4A3* reference sequence LRG_230 (NM_000091.4), *COL4A4* reference sequence LRG_231 (NM_000092.4) and COL4A5 reference sequence LRG_232 (NM_000495). To select for candidate modifiers genes after a first filtering step, we obtained for each samples an average of 85 likely protein-affecting variants (frameshift or in-frame insertions/deletions, stopgain/stoploss variants, splicing changes and missense variants predicted to be damaging for at least 2/6 employed bioinformatic tools and with a CADDphred ≥15), among which we selected for quality (depth ≥ 20X and phred quality score ≥ 40) and for MAF < 0.01 or unknown frequency on dbSNP151 only variants lying in candidate genes encoding for the extracellular matrix, basement membrane and slit diaphragm or podocyte cytoskeleton.

### Sanger sequencing and segregation analysis

Pathogenic variants identified in probands were confirmed by Sanger sequencing using the PE BigDye Terminator Cycle Sequencing Kit on an ABI Prism 3130 analyser (Applied Biosystems). The version 4.9 of Sequencher Software (Genes Code Corporation, Ann Arbor, Michigan, United States) was used for sequence analysis. Sanger sequencing was also used to determine whether the pathogenic variants were present in family members for whom genomic DNA was available. Genotypes of pedigrees were examined to assess genotype-phenotype correlation.

## Results

### Family 1: Clinical features

**Fig. 1:**
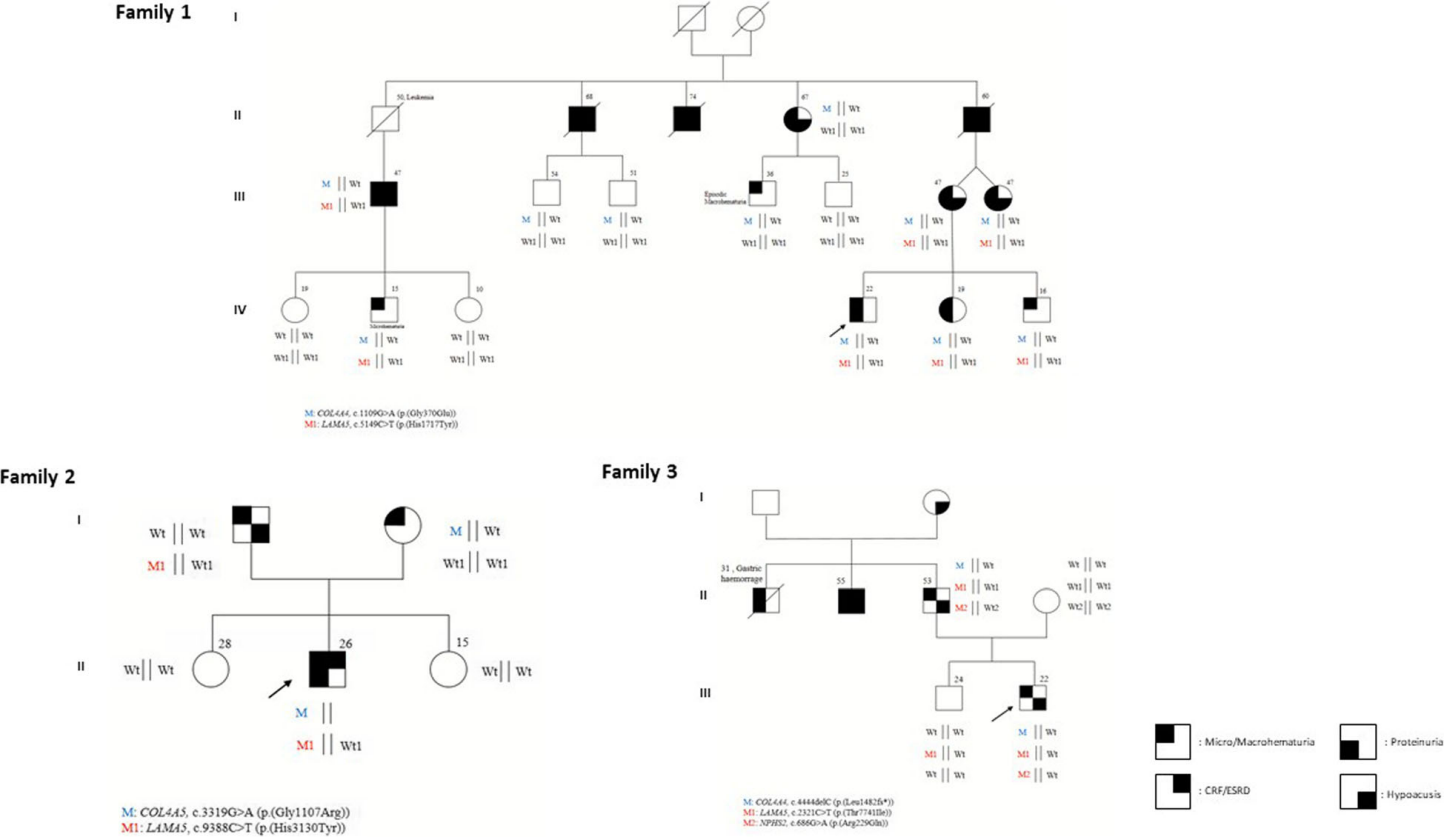
Families pedigrees. Pedigree of the families characterized at a transcriptional level are depicted and segregation of the mutated allele/s is reported

The proband (IV;4) (Fig. 1, Family 1), a 22 years-old male, presented at our outpatient clinics at 16 years of age with constant microhematuria and proteinuria (0,3 g/dl). Kidney biopsy examination revealed thinning of the glomerular basement membrane. Ophthalmoscopic evaluation showed a normal *fundus oculi*, audiometric evaluation was in the range of normality. At the anamnestic history, the parents reported macrohematuria episodes since he was 11 years old. The younger sister (IV;5) and brother (IV;6), 13 and 10 years old, respectively, at the time of the first visit displayed microhematuria with intermittent proteinuria identified in the younger sister in some of the several of the 15 urine exams viewed over the time. His mother (III;6) and her twin sister (III;7), presented with microhematuria and proteinuria as well. Audiometric evaluation revealed in both cases a bilateral neurosensorial hearing loss. The maternal grandfather (II;5) reported microhematuria and proteinuria in association with the appearance of bilateral kidney cysts at around forty-five years of age with progression towards end stage renal disease (ESRD) at around 50 years of age. For such reason he underwent kidney transplantation. Bilateral neurosensorial hypoacusis was also documented. The mother of the proband referred that two paternal uncles (II;2 and II;3) developed end stage renal disease at around 45 years of age. We evaluated the sister of the proband’s grandfather (II;4) when she was 62 years old and she presented with microhematuria, proteinuria and bilateral hearing loss. Out of the two sons, the oldest (III;4), 36 years old, presented with microhematuria after physical activity while the youngest (III;5), 25 years old, showed neither microhematuria nor proteinuria. Two sons of a proband’s maternal uncle (III;2 and III;3) who died for renal failure were evaluated at the age of 54 and 51 years; urine exams were negative for persistent microhematuria. An additional cousin of the proband’s mother (III;1), later evaluated in our outpatient clinics, presented with microhematuria, proteinuria and moderate renal failure started at the age of 40 years for which he currently undergoes dialysis. Kidney ultrasound identified a unilateral renal cyst. No audiometric evaluation was performed. His 15 years old son (IV;2) presented with isolated microhematuria.

#### Family 1: Molecular findings

Using a whole exome sequencing approach we identified, in the proband, a heterozygous pathogenic *COL4A4* mutation (exon 19:c.1109G > A:p.(Gly370Glu)) in line with an autosomal dominant pattern of transmission. A thorough data examination led to identify a hypomorphic variant in *LAMA5* gene (exon 39:c.5149C > T:p.(His1717Tyr)). The CADDPhred of the variant is 18,73, and according to the ExAc genomes database it has been previously found in European populations with a frequency of 0,07/119,590 (rs875379) (Table 1). The combination of the two *COL4/LAMA5* variants was identified in all the severely affected family members while the single pathogenic *COL4A4* mutation was found both in the first cousin (III;4) presenting with just episodic microhematuria at the age of 26 years and in two asymptomatic individuals (III;2 and III;3), evaluated at the age of 50 years, allowing us to conclude that the *COL4A4* mutation is not sufficient by itself for the phenotype to be displayed. An additional asymptomatic cousin (III;5) did not harbor any of the two mutations. These findings are in line with a digenic pattern of transmission as also suggested by the average age of ESRD onset in all the affected family members.

**Table 1:** Clinical characteristics and identified variants in three probands with Alport syndrome and their family members

Family no. and the individual pedigree ID	Age (years)	Mutation	Model	Microhematuria	Macrohematuria	Proteinuria	Creatinine	eGFR	Hearing deficiency	Visual deficiency	Ultrastructural microscopy	Mutations in modifier genes
Family 1 (IV; 4)	22	c.1109G > A (p.(Gly370Glu)) COL4A4	ADAS	Persistent	–	0,3 g/dL	–	–	–	–	GBM thinning	c.5149C > T (p.(His1717Tyr)) LAMA5 + [=]
Family 1 (IV; 5)	19	c.1109G > A (p.(Gly370Glu)) COL4A4	ADAS	Present	–	0,09 g/24 h	0,74 mg/dL	122,8 ml/min	–	–	–	c.5149C > T (p.(His1717Tyr)) LAMA5 + [=]
Family 1 (IV; 6)	16	c.1109G > A (p.(Gly370Glu)) COL4A4	ADAS	Present	–	0,08 g/24 h	0,57 mg/dL	152 ml/min	–	–	–	c.5149C > T (p.(His1717Tyr)) LAMA5 + [=]
Family 1 (III; 6)	47	c.1109G > A (p.(Gly370Glu)) COL4A4	ADAS	Persistent	–	Intermittent	–	–	Low frequency hypoacusia	–	–	c.5149C > T (p.(His1717Tyr)) LAMA5 + [=]
Family 1 (III; 7)	47	c.1109G > A (p.(Gly370Glu)) COL4A4	ADAS	Present	–	Intermittent	0,54 mg/dL	112,4 ml/min	Hypoacusia	–	–	c.5149C > T (p.(His1717Tyr)) LAMA5 + [=]
Family 1 (II; 4)	67	c.1109G > A (p.(Gly370Glu)) COL4A4	ADAS	Persistent	Persistent	Intermittent	0,9 mg/dL	82,5 ml/min	Bilateral Hypoacusia	Opalescence in the nucleus of the crystalline	–	–
Family 1 (III; 4)	36	c.1109G > A (p.(Gly370Glu)) COL4A4	ADAS	Absent	Absent	–	Absent	Absent	Absent	Absent	–	–
Family 1 (III; 5)	24	Absent	ADAS	Absent	Absent	–	–	–	–	–	–	–
Family 1 (III; 2)	54	c.1109G > A (p.(Gly370Glu)) COL4A4	ADAS	Absent	Absent	Absent	Absent	Absent	Absent	Absent	–	–
Family 1 (III; 3)	51	c.1109G > A (p.(Gly370Glu)) COL4A4	ADAS	Absent	Absent	Absent	Absent	Absent	Absent	Absent	–	–
Family 1 (III; 1)	47	c.1109G > A (p.(Gly370Glu)) COL4A4	ADAS	Absent	Absent	Intermittent	1,06 mg/dL	–	Absent	Absent	n/a renal cystis at the ultrasound	c.5149C > T (p.(His1717Tyr)) LAMA5 + [=]
Family 1 (IV; 1)	19	Absent	ADAS	Absent	Absent	Absent	Absent	Absent	Absent	Absent	–	–
Family 1 (IV; 2)	15	c.1109G > A (p.(Gly370Glu)) COL4A4	ADAS	Absent	Absent	Absent	Absent	Absent	Absent	Absent	–	c.5149C > T (p.(His1717Tyr)) LAMA5 + [=]
Family 1 (IV; 3)	10	Absent	ADAS	Absent	Absent	Absent	Absent	Absent	Absent	Absent	–	–
Family 2 (II; 2)	26	c.3319G > A (p.(Gly1107Arg)) COL4A5	XLAS	Present	Present in childhood	1,71 g/24 h	1,30 mg/dL	75,8 ml/min	Absent	Absent	Thickening, thinning, podocytes foot processes fusion	c.9388C > T (p(His3130Tyr)) + [=] LAMA5
Family 2 (I; 2)	50	c.3319G > A (p.(Gly1107Arg)) COL4A5	XLAS	Present	Absent	–	–	–	Absent	Absent	No ultrastructural lesions	–
Family 2 (I; 1)	53	None	n/a	Present	Absent	–	–	–	Monolateral hypoacusia	Absent	n/a	c.9388C > T (p(His3130Tyr)) + [=] LAMA5
Family 3 (III; 2)	22	c.4444delC (p.(Leu1482Trpfs*70)) COL4A4	ADAS	Persistent	Absent	–	0,61 mg/dL	1443,8 ml/min	Mild bilateral hypoacusia	Absent	Thickening, thinning, podocytes foot processes retraction, podocytes buldging	c.686G > A (p.(Arg229Gln)) NPHS2; c.2321C > T (p.(Thr774Ile)) + [=] LAMA5
Family 3 (II; 3)	52	c.4444delC (p.(Leu1482Trpfs*70)) COL4A4	ADAS	Present	Absent	0,5 g/24 h	0,80 mg/dL	102 ml/min	Mild bilateral hypoacusia	NA in Clinical Folder	n/a	c.686G > A (p.(Arg229Gln)) NPHS2; c.2321C > T (p.(Thr774Ile)) + [=] LAMA5

Mutations were all named in accordance with the standard nomenclature guidelines proposed by the Human Genome Variation Society (http://www.hgvs.org). Nucleotide numbering reflects cDNA numbering with + 1 corresponding to the A of the ATG translation initiation codon in the reference sequence (COL4A5, RefSeq NM_000495.4; COL4A4, RefSeq NM_000092; COL4A3, RefSeq NM_000091; LAMA5 RefSeq NM_005560; NPHS2, NM_014625)

### Family 2: Clinical features

The proband (Fig. 1, Family 2) came for the first time to our outpatient clinics when he was 17 years old. He presented with severe microhaematuria since he was 18 months old. The anamnestic history was positive for macrohaematuria during febrile episodes. Mild proteinuria, in the range of 400 mg/24 was detected since he was 13 years. Currently he is 26 years old. He displays proteinuria in the range of 1.71 g/24 h and serum creatinine and creatinine clearance in the upper range of normality (1,30 mg/dl and 105 cc/min, respectively). For this reason, he is actually under treatment with RAAS-inhibitors. Kidney biopsy examination revealed areas of thinning and thickening of the glomerular basement membrane along with podocytes foot processes fusion, compatible with clinical diagnosis of ATS. Ophthalmoscopic evaluation showed a normal *fundus oculi*, audiometric evaluation was normal as well. The proband’s father, currently 53 years old, presented microhematuria. His mother, 50 years old, reported microhematuria since she was teenager. She never presented with proteinuria. Audiometric evaluation was negative. Kidney biopsy did not reveal any ultrastructural alteration.

### Family 2: Molecular findings

Using a whole exome sequencing approach we identified, in the proband, a maternally inherited heterozygous pathogenic *COL4A5* mutation (exon 37:c.3319G > A:p.(Gly1107Arg)) in line with an X-linked pattern of transmission. A thorough data examination led to identify a hypomorphic variant in *LAMA5* gene (exon 69:c.9388C > T:p.(His3130Tyr)) The CADDPhred of the variant is 10,66 and in European populations, the *LAMA5* variant has been found with a frequency of 0.00014 (rs201154340) (Table 1). The *LAMA5* variant was also identified in the proband’s father who presented with microhematuria in line with a digenic pattern of transmission as also suggested by the biparental contribution.

### Family 3: Clinical features

The proband (Fig. 1, Family 3) came to our outpatient clinics when he was 15 years old. He presented with constant microhematuria since he was 7 years old, no proteinuria was detected in any of the urine exams in a time span of 8 years. Kidney biopsy examination revealed areas of thinning and thickening of the glomerular basement membrane along with podocytes foot processes retraction and simplification and podocytes bulging, compatible with clinical diagnosis of ATS. Ophthalmoscopic evaluation showed a normal *fundus oculi*, audiometric evaluation displayed a mild bilateral hypoacusia since he was 12 years old. The proband’s brother was asymptomatic. His father, 50 years old, reported microhematuria since he was teenager. He did not presented with proteinuria. Audiometric evaluation was positive for mild bilateral hypoacusia. The anamnestic history was positive for microhematuria and proteinuria in a paternal uncle who died at 31 years of age for a gastric haemorrhage. It was also positive for bilateral hypoacusis and ESRD in a second paternal uncle who required kidney transplantation at the age of 40 years.

#### Family 3: Molecular findings

A whole exome sequencing approach allowed us to identify, in the proband, a heterozygous pathogenic *COL4A4* mutation (exon 46:c.4444del:p.(Leu1482Trpfs*70)) in line with an autosomal dominant pattern of transmission. Data examination also led to identify two hypomorphic variant, one in *LAMA5* gene (exon 18:c.2321C > T:p.(Thr774Ile)) and the other in *NPHS2* (exon5:c.686G > A (p.(Arg229Gln)). The CADDPrhed of the variants are 23,2 for *LAMA5* and 18,73 for *NPHS2*, respectively (Table 1). According to the ExAc genomes database the *LAMA5* has been previously found in European populations with a frequency of 0.001867 (rs145721906) and reported as possibly damaging. According to SpliceSiteFinder-like and Human Splicing Finder the variant, lying at − 3 nt from the GT of the 5′ splice site consensus sequence, could also have an impact on splicing through an alteration of the splice donor site or an alteration of ESS and ESE sites, respectively. However, given the low-level expression of LAMA5 transcript in peripheral blood lymphocytes further mRNA studies have not been performed. The *NPHS2* variant has been found with a frequency of 0,03 according to the ExAc database; however it has been previously reported as likely pathogenic (rs61747728) and associated with nephrotic syndrome [17]. The combination of the *COL4/LAMA5/NPHS2* variants was also identified in the father while the unaffected brother only shared the *LAMA5* mutation. Unfortunately the proband’s uncle denied his consent to perform molecular testing.

All the clinical features, mutations in COL4 causative genes and in the modifier genes, for the three families, are reported in Table 1.

All the electropherograms of the mutations in COL4 causative genes and in the modifier genes, for the three families, are reported in Additional file 1.

## Discussion

ATS is one of the most common inherited form of glomerulopathy, often associated with deafness and ocular lesions. Four clinical criteria, which include positive family history of hematuria with or without chronic renal failure, unique ultrastructural changes in the GBM, and extrarenal manifestations for the diagnosis of ATS were proposed by Flinter in 1988 [18]. Genetic testing is always recommended and is the only tool able to confirm the diagnosis and define the inheritance pattern. Employing a targeted next-generation multigene panel in a clinically heterogeneous nephropathy such as ATS, we have recently been able to define a new digenic pattern of inheritance with pathogenic mutations in two collagen IV genes. However, daily clinical practice prompt us to realize the existence of families in which a collagen-related genes digenic pattern of inheritance is not sufficient to justify the clinical severity, the intrafamilial variable expressivity and sometimes the histopathological findings. Recent works have suggested a role of other genes in exacerbating clinical phenotype in ATS patients with *COL4A5* mutations [19]. Recently an association of *LAMA5* and *COL4A5* variants has been described in four family members presenting with microhematuria and proteinuria and progressive ESRD in the spectrum of ATS [15].

For the first time, here we describe three families, two with an apparent autosomal dominant ATS and one with an X-linked pattern of transmission, in which a pathogenic mutation in *COL4A4* or *COL4A5* cosegregate with a likely damaging variant in *LAMA5*. Notably, in family 1, a very large informative family, the combination of *LAMA5/COL4A4* variants was observed in all severely affected family members, while the single *COL4A4* variant was detected in one individual with episodic microhematuria and in two asymptomatic older cousins, suggesting, a digenic inheritance rather than a modifier effect, according to which the coexistence of both variants is necessary in order to display all the clinical criteria for ATS diagnosis. We have previously provided clear evidence of the existence of an AD form of Alport syndrome [12]. Here we like to introduce the concept of a mutation type-dependent pattern of transmission based on which severely damaging COL4a3 and a4 chains mutations in heterozygous state are sufficient to determine an autosomal dominant ATS while hypomorphic mutations need to cosegregate together or in association with variants in other genes of the extracellular matrix or podocyte cytoskeleton in order for a fully penetrant phenotype to develop.

Previous works have shown that the glomerular filtration barrier integrity is ensured by laminin alpha5. Podocytes specific inactivation of Lama5 in mice, results in varying degrees of proteinuria and progression to nephrotic syndrome likely related to a thickening of the GBM associated with podocyte foot processes disruption [20]. This is in line with the histopathological findings of the probands in family 2 and 3 who displayed thickening and thinning of the GBM associated with podocytes foot processes retraction or fusion which is rarely observed in collagen 4 genes-related ATS and that should be regarded as a pathognomonic sign of *LAMA5* variants coinheritance. Proband’s father in family 2, harboring the single *LAMA5* mutation displays constant microhematuria suggesting that damaging *LAMA5* mutations in a heterozygous state could alone explain familial hematuria. In line with previous reports [15] renal cysts were also observed in family 1 affected members who harbor the combination of *LAMA5/COL4A4* mutations. These findings urge the need to investigate for a *LAMA5* mutation in all ATS individuals in which kidney biopsy is positive for the presence of renal cysts and podocytes foot processes fusion or retraction.

In family 3 affected individuals also harbored a *NPHS2* variant. The *NPHS2* variant c.686G > A (p.(Arg229Gln)) (rs61747728), is reported as one of the most important predictive factors for steroid-resistant nephrotic syndrome and FSGS [21]. Some studies have suggested that a recessive inheritance of the variant is enough to determine the phenotype [22], while others have suggested that the p.(Arg229Gln) is pathogenic only when it is inherited in a compound heterozygote state with another *NPHS2* mutation or in combination with other variants [23]. Our data suggests that in a heterozygote state, this allele contributes to an oligogenic form of disease in which any single gene plays a different role sometimes distinct among the affected individuals. Due to the young age of the proband at the moment of the clinical evaluation we cannot exclude the future onset of proteinuria as reported in the paternal uncle with whom he likely shares the combination of *COL4A4* mutation with at least one of the other two variants. However, it is also plausible to think that in each family member the existence of different trans-splicing factors would determine a variable effect of the *LAMA5* splicing variant on its own transcript leading to a distinct clinical expressivity.

The mean age of onset of ESRD in our group of patients harboring a combination of *COL4*/*LAMA5* variants (II;2, II;3, II;4; II;5 in family 1 and II;2 in family 2) is 45 years, in line with previously reported data about collagen 4 chains-related digenic inheritance [13, 14], suggesting to suspect a digenic inheritance when the age of the renal failure onset is intermediate between an autosomal dominant and a recessive form. Ultimately, these data highlight the need for a next generation sequencing approach broadened to include non- collagen genes in order to better define clinical severity and recurrence risk for other family members. Definitely, a larger study which compares patients with only a collagen 4-gene mutation and patients harboring a combination of collagen 4/non-collagen genes variant is needed in order to prove an additive effect of the identified alterations.

## Conclusions

In conclusion, these results reinforce the existence of a digenic form of ATS umasking the role of non-collagen genes, essential for the GBM formation and stabilization. Unique pathognomonic findings from kidney biopsies such as renal cysts and podocytes foot processes fusion or retraction should be regarded as hallmark of a *LAMA5*/*COL4* variants coinheritance. Moreover, these findings change the clinical management of patients with Alport syndrome highlighting the importance of a NGS approach broadened to include genes of the extracellular matrix (basement membrane and slit diaphragm) or podocyte cytoskeleton.

## Additional file

Additional file 1: Electropherograms of the mutations in *COL4* and in the modifier genes. Electropherograms of the mutations in *COL4* causative genes and in the modifier genes, for the three families. (JPG 144 kb)

## Abbreviations

ADAS: Autosomal Dominant Alport Syndrome
ARAS: Autosomal Recessive Alport Syndrome
ATS: Alport Syndrome
GBM: Glomerular Basement Membrane
TBMN: Thin Basement Membrane Nephropathy
WES: Whole-Exome Sequencing
XLAS: X-Lniked Alport Syndrome
