# Supplementary figures and images for: Non-collagen genes role in digenic Alport syndrome
Source: BMC Nephrol. 2019 Feb 26;20:70. doi: 10.1186/s12882-019-1258-5 (PMC6391820; doi:10.1186/s12882-019-1258-5)

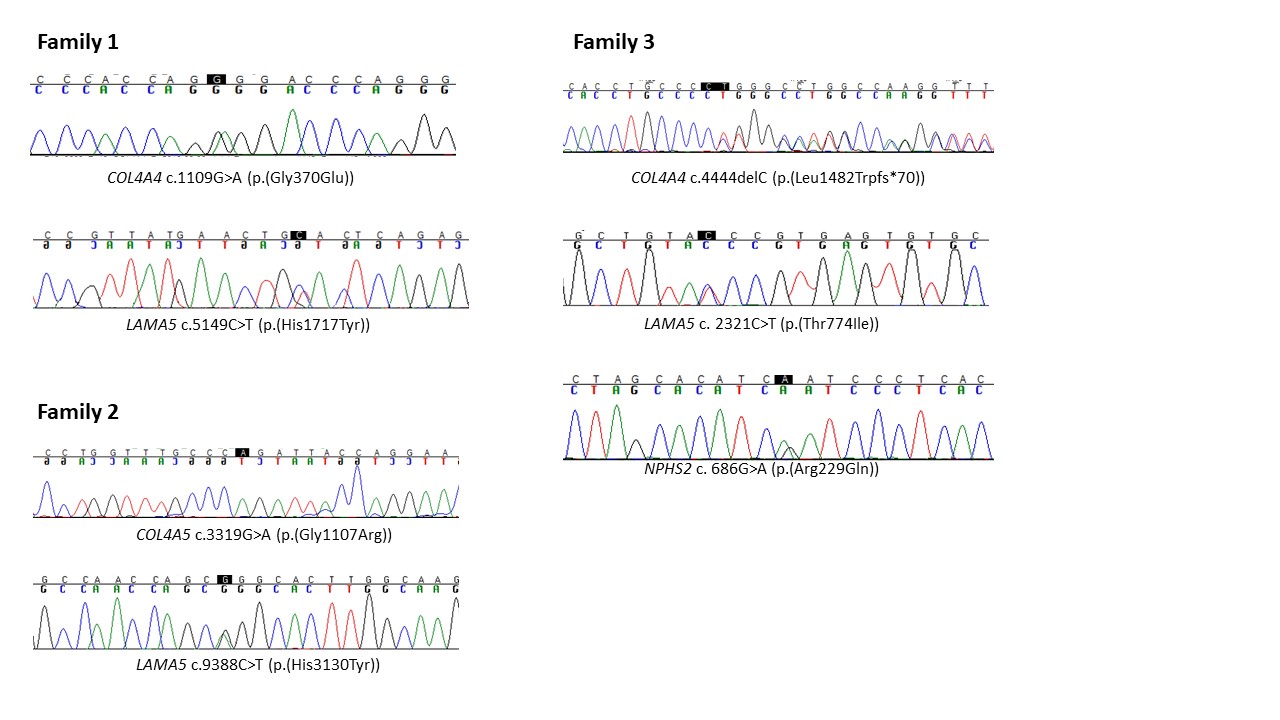

Supplement: Supplementary file 1 — Electropherograms of the mutations in COL4 and in the modifier genes. Electropherograms of the mutations in COL4 causative genes and in the modifier genes, for the three families. (JPG 144 kb) [file 12882_2019_1258_MOESM1_ESM.jpg]
